# An Efficient Differential Privacy-Based Method for Location Privacy Protection in Location-Based Services

**DOI:** 10.3390/s23115219

**Published:** 2023-05-31

**Authors:** Bo Wang, Hongtao Li, Xiaoyu Ren, Yina Guo

**Affiliations:** 1 School of Electronic Information Engineering, Taiyuan University of Science and Technology, Taiyuan 030024, China; mophiebo@126.com; 2 College of Mathematics and Computer Science, Shanxi Normal University, Taiyuan 030039, China; lihongtao7758@163.com (H.L.); 19935646006@163.com (X.R.)

**Keywords:** location-based services, location privacy protection, differential privacy, cluster model

## Abstract

Location-based services (LBS) are widely used due to the rapid development of mobile devices and location technology. Users usually provide precise location information to LBS to access the corresponding services. However, this convenience comes with the risk of location privacy disclosure, which can infringe upon personal privacy and security. In this paper, a location privacy protection method based on differential privacy is proposed, which efficiently protects users’ locations, without degrading the performance of LBS. First, a location-clustering (L-clustering) algorithm is proposed to divide the continuous locations into different clusters based on the distance and density relationships among multiple groups. Then, a differential privacy-based location privacy protection algorithm (DPLPA) is proposed to protect users’ location privacy, where Laplace noise is added to the resident points and centroids within the cluster. The experimental results show that the DPLPA achieves a high level of data utility, with minimal time consumption, while effectively protecting the privacy of location information.

## 1. Introduction

With the rapid development of mobile intelligent devices and location technology, various types of location-based services (LBS) applications have brought convenience to people’s lives. While enjoying these convenient services, mobile users need to provide specific location information such as the nearest subway station, hospital, or bank. However, user location information is closely linked to personal living habits, health, economic conditions, and other private information [1], which can be used to mine, analyze, or infer users’ private information. In recent years, several high-profile cases of location privacy leaks have occurred that resulted in serious consequences, including (1) Stalking and physical harm: The application (app) Girls Around Me was found to collect user information from Facebook and Instagram to create a map of the locations of women nearby, without their knowledge or consent. This could potentially lead to stalking or even murder. (2) Identity theft: Location sharing and geotagging in social media apps such as Snapchat and Instagram can reveal personal information about an individual’s location, which can lead to identity theft, credit card fraud, and phishing attacks. (3) Theft: Location-sharing through social media platforms can also be used by thieves to determine when someone is away from home, making them a potential target for theft. So, users can experience serious consequences if they provide precise location data through LBS [2]. To avoid these problems, there is a pressing need to protect users’ location privacy.

Existing location privacy protection technologies include k-anonymity, l-diversity, and differential privacy (DP). k-anonymity and l-diversity generalize a user’s real location into an area to achieve location protection. However, this only protects users’ privacy to a certain extent and cannot prevent homogeneous attacks [3] and background knowledge attacks [4]. Qian et al. proposed a privacy protection model that can prevent background knowledge attacks and provide a quantitative evaluation method, namely differential privacy [5]. In recent years, LBS protection algorithms based on DP have become a focus of research, but they can fall short of effectively preventing continuous location tracking and identification [6].

To address these challenges, we are committed to developing a method that makes it difficult for attackers to infer a user’s exact location (protect location privacy) and sensitive attributes (protect query privacy) from query sequences, regardless of how much prior knowledge they possess. At the same time, the method ensures the accuracy of each LBS query, without any additional overhead, that is, the final query results obtained by a user remain the same even after privacy protection is added. Based on these concerns, we propose a privacy protection method based on differential privacy and L-clustering that is suitable for continuous location from the perspective of the above-mentioned goals. This method not only guarantees strong privacy but also maximizes data utility. The main contributions of this study are as follows:

(1) According to the distance and density between locations, an L-clustering algorithm is proposed to find the centroid of each cluster and replace all the locations within the cluster. Moreover, the continuous locations are divided into different regions of interest (ROIs) based on the user’s access frequency in different locations. This method can reduce the computation burden of differential privacy.

(2) A differential privacy-based location privacy protection algorithm (DPLPA) is proposed. The resident point is extracted based on whether the user’s access time, access frequency, and location contain sensitive information. In addition, a privacy budget is allocated to the resident point and cluster centroid. At the same time, Laplace noise is added to the resident point and cluster centroid to protect location privacy.

(3) Considering the user’s privacy preferences, different privacy budgets are allocated to different resident points, and the range of false location generation acceptable to users is determined to generate ROIs with higher utility. Theoretical analysis and experimental results show that DPLPA can effectively protect location privacy in LBS.

The rest of the paper is organized as follows. Section 2 introduces the related works on privacy protection in LBS and the related major challenges. In Section 3, we provide definitions of differential privacy, system structures, and the threat model of the algorithm. Section 4 describes the proposed L-clustering algorithm and DPLPA and theoretically analyzes the algorithms in terms of security, time complexity, the degree of privacy protection, and data utility. In Section 5, we carry out simulation experiments to evaluate the clustering accuracy, degree of privacy protection, data utility, and running time of each algorithm. Finally, we conclude our paper and provide some future perspectives in Section 6.

## 2. Related Works

Many studies have proposed methods for LBS privacy protection involving k-anonymity, l-diversity, and differential privacy [7,8,9]. Zhang et al. [10] proposed a novel method of location privacy protection based on geographic semantics and ensuring k-anonymity. In this method, a candidate set is constructed using the maximum and minimum distance multi-center clustering algorithm, and the virtual location results are generated based on semantic similarity. Xing et al. [11] proposed a modified privacy protection scheme based on double k-anonymity that hides users’ locations and request information. Tian et al. [12] constructed a semantic and trade-off-aware location privacy protection mechanism (STA-LPPM) in which the multi-objective particle swarm optimization algorithm is used to generate an optimal anonymous set, achieving a balance between privacy protection and quality of service. A blockchain-enabled framework for peer-to-peer (P2P) energy trading was designed in [13], and an anonymous proof-of-location algorithm was proposed that allows clients to choose their trading partners without revealing their real locations. Zheng et al. [14] employed a dynamically adjustable k-anonymity (DAK) algorithm and a dynamical location privacy protection (DLPP) algorithm based on virtual locations in which sequences are disturbed by adding and deleting moving points. However, the effectiveness of combining l-diversity and k-anonymity is limited by data distribution and background knowledge attacks. As a result, the level of privacy protection cannot be guaranteed.

In addition to the above methods, there are models of LBS privacy protection that consist of a location tree, Markov model, and clustering. The main idea behind a location tree is to construct a tree structure based on certain rules. The prefix tree and DP [15] are used to protect the privacy of the trajectory data and the nodes of the tree are used to store the trajectory segments. Li et al. [16] established a hierarchical tree structure based on location attributes and proposed an attribute-aware privacy-preserving scheme for LBS. In addition, a Markov model is used to simulate the temporal correlation between a user’s real location and the prediction of the next possible location based on the transition probability of each location. Yuan et al. [17] proposed a new location privacy protection method for a Cloud-of-Things system in which a Markov model is used to analyze users’ mobile behavior. The proposed location-hiding algorithm meets users’ privacy requirements by expanding the sizes of areas. Partovi et al. [18] modeled a Markov decision process and introduced a new location privacy measurement method to ensure that a user’s specified privacy level could be achieved over an infinite time range. Yang et al. [19] used k-anonymity to enhance privacy protection and clustering technology to group users by learning their trajectory data. A graph-based trajectory data representation model [20] was proposed in which the similarity between trajectories is calculated using a measurement method based on edges and nodes and similar trajectories are clustered and identified based on their paths. Clustering can capture users’ activity patterns over a certain period and can remove locations with low access frequencies, so it is very flexible.

Differential privacy is a useful method due to its good privacy protection performance. In addition, it can efficiently prevent inference attacks by adding random noise to the original query results (adding or deleting some of the data in the datasets does not affect the query results). Therefore, it is difficult for attackers to infer real data through the use of multiple queries, thus achieving privacy protection. Stephanie et al. [21] used DP technology to protect location data. In this method, random noise is added to confuse a user’s location, and the centroids of the clusters are gathered on a cloud server to generate the final cluster. This method provides an efficient privacy-preservation solution for location-based data-stream processing. Hu et al. [22] considered the personalized security requirements of different users to achieve location protection based on users’ historical global positioning system (GPS) trajectory data and the natural attributes of locations. However, it has a massive computational load, and the accuracy of the user sensitivity evaluation is poor. Wang et al. [23] proposed a privacy-protected social tie mining (P-STM) method, which can identify social connections from users’ daily trajectories, and offered an indicative dense region to calibrate personal daily trajectories. In addition, a clustering analysis method for spatiotemporal sequence data was proposed in [24]. This method provides a basis for privacy protection by constructing continuous time regions and includes a data publishing mechanism that can prevent inferential attacks. However, this mechanism mainly distributes the offline group location data and cannot update other relevant information. A new framework (PrivSem) was presented in [25], which combines k-anonymity, l-semantic diversity, and DP. It guarantees location privacy, but setting a non-sensitive location as a sensitive location can increase the cost of privacy protection.

The literature review is summarized in Table 1.

## 3. Preliminaries

### 3.1. Definitions

**Definition** **1.** *(Adjacent Datasets). Suppose that the datasets D have the same attribute structures and there is only one record that is different between them. If $\left|D\Delta D^{\prime }\right|=1$, the datasets D and $D^{\prime }$ are called adjacent datasets.*

*Let d be a positive integer and $f:D\to R^{d}$ be a function. The function sensitivity represented by Δf has the following definition:*

(1) $$\Delta f=\max{∥f\left(D\right)-f\left(D^{\prime }\right)∥}_{1}$$

*where $∥.∥$ is the Manhattan distance in this paper.*

**Definition** **2.** *(Differential Privacy). There is a random algorithm A and all possible outputs of A are $P_{A}$. For any two neighboring datasets D and $D^{\prime }$ and any subset $S_{A}$ of $P_{A}$, algorithm A satisfies the following conditions:*

(2) $$\mathrm{Pr}\left[A\left(D\right)\in S_{A}\right]\leq e^{\varepsilon }\mathrm{Pr}\left[A\left(D^{\prime }\right)\in S_{A}\right]$$

(3) $$\forall t\in Range\left(A\right),D≃D^{\prime }:\frac{\mathrm{Pr}[A(D)]}{\mathrm{Pr}[A\left(D^{\prime }\right)]}\leq e^{\varepsilon }$$

*Algorithm A satisfies the ε-differential privacy, where the parameter ε is the privacy budget.*

**Definition** **3.** *(Privacy Budget). The privacy budget ε reflects the level of privacy protection.*

(4) $$\varepsilon \geq \max\left(\ln\frac{\mathrm{Pr}\left[A\left(D\right)\right]}{\mathrm{Pr}\left[A\left(D^{\prime }\right)\right]}\right)$$

*The larger the ε, the higher the data utility and the lower the level of privacy protection. On the contrary, the smaller the ε, the lower the data utility and the higher the level of privacy protection.*

**Definition** **4.** *(Laplace Mechanism). Given the datasets D, the random algorithm $M\left(D\right)=f\left(D\right)+Y$ provides ε-differential privacy protection, where $Y∼L\mathrm{ap}\left(\Delta f/\varepsilon \right)$ is the random noise and obeys the Laplace distribution with the scale parameter Δf/ε. The function is shown in Equation (5):*

(5) $$A_{f}=f\left(D\right)+Lap\left(\frac{\Delta f}{\varepsilon }\right)\;\;\;\;\;\;satisfies\;\;\;\;\;\;\varepsilon -DP$$

*The Laplace mechanism realizes differential privacy by adding Laplace noise to the query results. Note that the location parameter is 0 and the scale parameter is P(b) of b. Then, the probability density function is calculated, as shown in Equation (6):*

(6) $$\mathrm{Pr}\left(\mu \right)=\frac{1}{2b}e^{-\frac{\left|\mu \right|}{b}},\;\mu =\left(r,\theta \right),\;\theta \in \left[0,2\pi \right]$$

*where r is the distance of $m_{0}$ from $m_{1}$, and θ is the angle that the lines $m_{0}$ and $m_{1}$ form with respect to the horizontal axis of the Cartesian system.*

**Definition** **5.** *(Region of Interest, ROI). Set the distance threshold to E. The continuous location $L_{1}=\left\{m_{1},m_{2},\ldots ,m_{n}\right\}$, $dis\left(m_{n},m_{n+1}\right)\leq E$. The region formed by the sequence of moving continuous locations from location $m_{l}$ to $m_{l+1}$ is the user’s ROI, where E is the maximum distance threshold required to form the ROI and $d\left(m_{n},m_{n+1}\right)$ is the distance between two locations.*

**Definition** **6.** *(Data Utility). Data utility is measured as shown in Equation (7):*

(7) $$U=\sqrt{\frac{\sum_{i\in R}\rho i-\rho i^{\prime }}{\left|R\right|}}$$

*where R is the number of clusters and ρ represents the density of each cluster.*

### 3.2. LBS System Model

**Figure 1:**
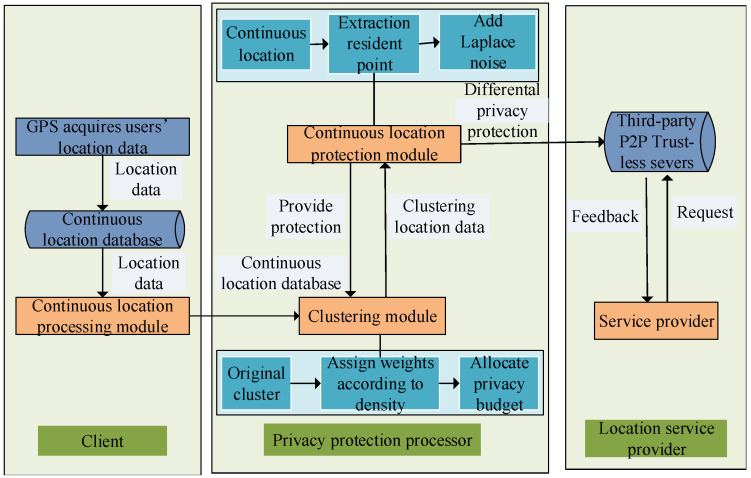
LBS system architecture.

The LBS system architecture of this paper is shown in Figure 1, which mainly includes the client, privacy protection processor, untrusted third-party server, and location service provider. The client obtains users’ location data through GPS and uploads this data to a location database. The privacy protection processor includes a clustering module and a continuous location protection module. The clustering module divides users’ location data into clusters based on distance and density. The continuous location protection module provides differential privacy protection. The untrusted third-party server is a peer-to-peer server. The location service provider provides query services for users and returns query results to users.

The system adopts a fully distributed architecture and a peer-to-peer network communication mode. All participants have the function of relay forwarding, which hides the communication participants within multiple network entities. In this way, the flexibility and reliability of anonymous communication are improved, the privacy of users is better protected, and robustness and invulnerability are superior to that of a traditional client/server (C/S) network [26]. However, there are still some hidden risks of privacy leakage in the release of users’ location information. To address this problem, we propose a continuous location protection method based on differential privacy in this paper. Firstly, a user’s location is simplified based on the location access frequency, which is obtained through GPS. Secondly, location data are clustered based on the distance and density between locations, and a clustering centroid is obtained. Finally, the resident points are extracted using the DPLPA, and Laplace noise is added to the resident points and centroids. The privacy-protected data are stored in the database for querying by the location service provider.

### 3.3. Threat Model

Attacker’s Capability. Homogeneous attacks take advantage of the fact that the values of sensitive attributes in a group of *k* records are the same. In this case, even if the data have been *k*-anonymized, an attacker can accurately predict the sensitive values of *k* records and easily obtain the desired information. A background knowledge attack is where an attacker can deduce privacy information with a high probability based on existing background knowledge, even if the sensitive attribute values in the k-anonymous group are different. Attackers can easily get the information they want.

Defender’s Knowledge and Capability. Differential privacy technology can effectively prevent the two above-mentioned types of attacks. According to Definition 2, even if certain personal information is in the k-anonymous group, the query results are all basically the same. An attacker is unable to determine whether someone’s information is in the query results, and the similarity of these results is controlled by the privacy budget ϵ.

**Figure 2:**
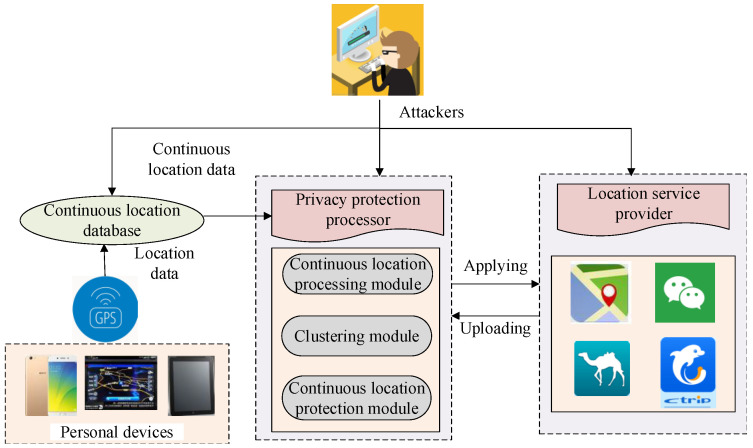
Threat model.

We assume that an attacker has arbitrary background knowledge, which can enable them to launch a background knowledge attack. We also assume that the attack can be a privacy attack from an untrusted third-party data collector. Users send their identities, locations, or hobbies to LBS providers to gain access to certain services, such as road congestion forecasts, traffic accident location reminders, nearby parking lots, etc. Once these LBS providers are attacked, users’ location data and other personal information can be leaked. Based on this assumption, a threat model is proposed, as shown in Figure 2.

## 4. Differential Privacy-Based Location Privacy Protection Method for LBS

### 4.1. Construction of Users’ Regions of Interest Based on Clustering

**Figure 3:**
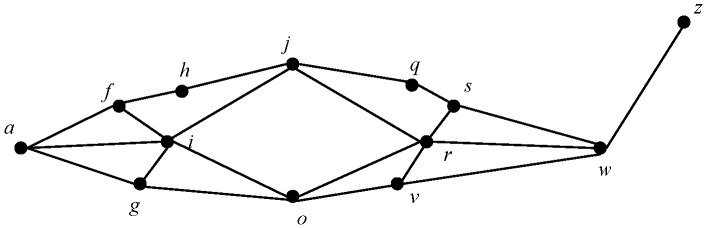
Users’ motion mode.

**Figure 4:**
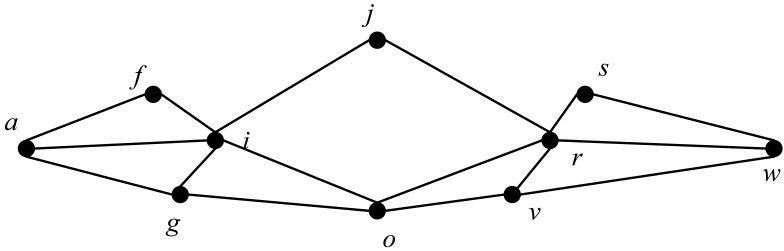
Motion mode after location restore.

To better protect the privacy of location data, firstly, a user’s location data are simplified, and their continuous positions over a period of time are recorded, as shown in Table 2. Secondly, according to the continuity of the positions over time, data on the user’s continuous positions are generated, and a position can appear multiple times in multiple groups of continuous positions, as shown in Figure 3. In the figure, the solid dot represents the location of the user, and the line between the two locations represents the user’s moving route. Finally, the user’s access times to each accurate location within the continuous positions are counted, and locations with access times below a threshold ξ are removed, as shown in Table 3. In this way, the number of continuous positions can be reduced, and the results are shown in Figure 4.

**Figure 5:**
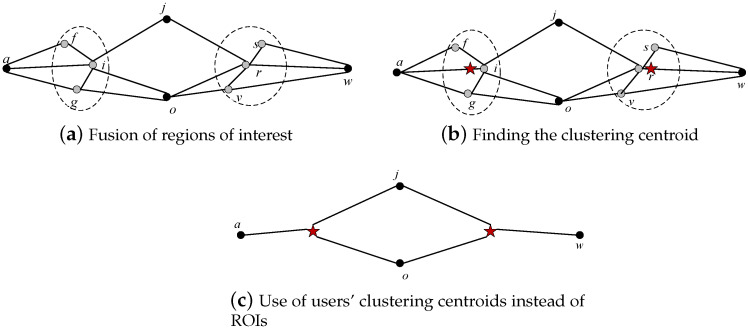
Fusion of continuous locations. The red star represents the centroid of a cluser.

For the reduced location data, it is necessary to construct ROIs. In the continuous locations, locations whose distances between locations are less than *E* are categorized into the same ROI, and the results are shown in the dotted circle in Figure 5a. The centroid of each ROI is determined and is represented by a red five-pointed star, as shown in Figure 5b. The centroid replaces other locations in the ROI to form new continuous location data, as shown in Figure 5c.

In order to address the problem of density-based spatial clustering of applications with noise (DBSCAN) [27], a continuous location-clustering algorithm (L-clustering) is proposed based on users’ ROIs. The pseudocode for this algorithm is shown in Algorithm 1. Firstly, calculate the distance between the location and the adjacent location for each location. If it is less than *E*, categorize the two locations into the same cluster; otherwise, they belong to different clusters. Then, mine users’ activity within a certain distance and use the centroid *c* of a cluster to represent this area. At the same time, other location points in this area are removed from the continuous location to avoid location redundancy.
**Algorithm 1:**   L-clustering algorithm.
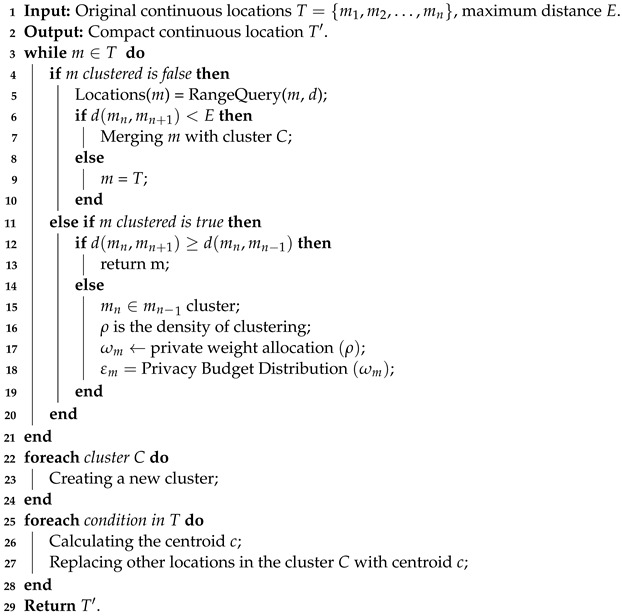


The L-clustering algorithm is used for clustering and dividing the densely distributed regions and consists of four steps. Lines 3 to 7 determine whether the current location *m* belongs to a cluster, query the distance between the current location and other locations, and compare the distance between them and the distance threshold *E*. If it is less than *E*, cluster *C* is formed. Otherwise, *m* is an independent location. Lines 8 to 12 determine the distance between $m_{n}$ and $m_{n-1}$ and the distance between $m_{n}$ and $m_{n+1}$ for the clustered location. If the distance between $m_{n}$ and $m_{n+1}$ is large, $m_{n}$ still belongs to the current cluster; otherwise, it belongs to another cluster. Lines 13–15 allocate a privacy budget to each cluster based on the density. Lines 19–20 calculate the centroid *c* of each cluster and use the centroid to replace other locations in the cluster to create a new continuous location as the publishing location.

### 4.2. Location Privacy Protection Algorithm Based on Differential Privacy

Aiming at addressing the problem of location privacy leakage, a differential privacy-based continuous location privacy protection algorithm (DPLPA) is proposed, its pseudo-code is shown in Algorithm 2. This algorithm extracts habitual residence and highly frequented access location points that contain sensitive user information and defines them as resident points. The pseudocode for this algorithm is shown in Algorithm 1. To determine the residence time, the duration between two places is considered. If $t\left(m_{i}\right)-t\left(m_{i-1}\right)\geq t_{time}$, it is defined as a time resident point. For the highly frequented access points, the access frequency of each location is considered. If $f\left(m_{j}\right)-f\left(m_{j-1}\right)\geq t_{fre}$, it is defined as a frequency resident point. For a location that contains users’ sensitive information, it is defined as a sensitive resident point. Finally, Laplace noise is added to the resident points.
**Algorithm 2:**   Differential Privacy-Based Location Privacy Protection Algorithm (DPLPA).
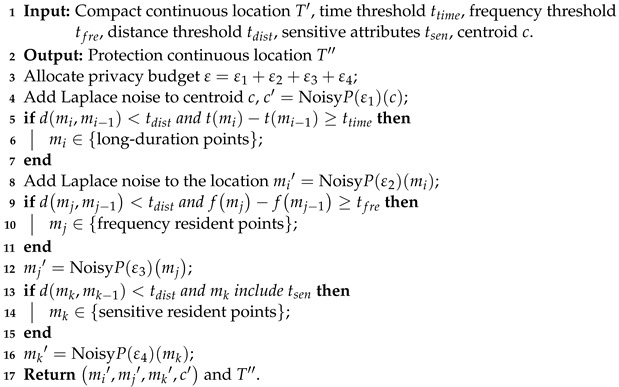


The primary task of the DPLPA is to extract the resident points and add Laplace noise that conforms to the differential privacy mechanism to the users’ simplified continuous location $T^{\prime }$, which includes four steps. Line 2 adds Laplace noise to the centroid. Lines 3–6 extract the time resident points and add Laplace noise to them. Lines 7–10 extract the frequency resident points and add Laplace noise to them. Lines 11–14 extract the sensitive resident points and add Laplace noise to them.

### 4.3. Theoretical Analysis

#### 4.3.1. Privacy Protection Analysis

The main reasons for adopting the differential privacy protection method in this paper are as follows:

(1) Differential privacy assumes that an attacker has complete background knowledge; therefore, it can efficiently prevent a background knowledge attack, even if an attacker knows all the information except for the original data;

(2) Differential privacy defines the privacy budget using a strict mathematical model, which ensures that the ratio of query results on adjacent datasets is less than or equal to $e^{\varepsilon }$.

Differential privacy can achieve privacy protection by adding random noise to the input data. In this study, users’ location data are considered numerical data so differential privacy technology is applicable. In the DPLPA, Laplace noise that obeys the distribution *P*(*b*) is added to the clustered data, which satisfies the differential privacy constraint. The proof is shown below.

It is proven that the probability density function $\mathrm{Pr}\left(\mu \right)=\frac{1}{2b}e^{-\frac{\left|\mu \right|}{b}}$ of the Laplacian mechanism is known. *x* and *y* represent two different positions and the probability density functions of Pr${}_{x}$ and Pr${}_{y}$ are $A_{m}\left(x,f,\varepsilon \right)$ and $A_{m}\left(y,f,\varepsilon \right)$ respectively. For a certain output value Z, there is:

(8) $$\frac{\mathrm{Pr}x(Z)}{\mathrm{Pr}y(Z)}=\prod_{i=1}^{k}\frac{e^{-\frac{\varepsilon |f(x)i-Zi|}{\Delta f}}}{e^{-\frac{\varepsilon |f(y)i-Zi|}{\Delta f}}}=\prod_{i=1}^{k}e^{\frac{\varepsilon (|f(y)i-Zi|-|f(x)i-Zi|)}{\Delta f}}\leq \prod_{i=1}^{k}e^{\frac{\varepsilon (\left|f(x)i\right|-|f\left(y\right)i|)}{\Delta f}}=e^{\frac{\varepsilon {∥f(x)-f(y)∥}_{1}}{\Delta f}}\leq e^{\varepsilon }$$

where ${∥\cdot ∥}_{1}$ represents the first-order normal form distance. According to the definition of differential privacy, the DPLPA satisfies the ϵ-differential privacy.

#### 4.3.2. Complexity Analysis

In this paper, the computation complexity involves the running times of the L-clustering algorithm and the DPLPA, and it is assumed that there are *n* records in the location data.

The L-clustering algorithm comprises four steps. First, the locations are divided into clusters based on the distance between the current location and its previous location and the current location and the following location; its time complexity is *O*(*n*). Second, based on the distance, the method can determine whether the clustering locations need to be reclassified, and its time complexity is *O*(2*n*). Third, the weight of each cluster based on its density and ϵ is calculated, and its time complexity is *O*(*n*). Fourth, the centroid *c* of each cluster is calculated and replaced with other locations in the cluster, and its time complexity is *O*(*n*). Therefore, the total time complexity is *O*(*n*) + *O*(*n*) + *O*(*n*) + *O*(*n*) ≈ *O*(*n*).

The DPLPA comprises four steps. First, Laplace noise is added to the centroid, and its time complexity is *O*(*n*). Second, the time resident points are extracted based on the time of the access location and Laplace noise is added; the time complexity is *O*(*n*). Third, the frequency resident points are extracted based on the access frequency of the location and Laplace noise is added; the time complexity is *O*(*n*). Fourth, the sensitive resident points are extracted based on whether users’ sensitive information is included in the location and Laplace noise is added; its time complexity is *O*(*n*). Therefore, the total time complexity is *O*(*n*) + *O*(*n*) + *O*(*n*) + *O*(*n*) ≈ *O*(*n*).

In summary, the total time complexity of the proposed approach is *O*(*n*) + *O*(*n*) ≈ *O*(*n*).

#### 4.3.3. Data Utility Analysis

Data utility refers to the difference between the processed data and the original data after adding Laplace noise. Data utility can be analyzed using Equation (7) in Definition 6. There are two main factors that can affect data utility, which are the number of clusters $\left|R\right|$ and the clustering density ρ. $\left|R\right|$ is inversely proportional to *U*, meaning that a higher number of clusters corresponds to a smaller *U* value and a greater data utility. This is because an increased number of clusters results in enhanced similarity between the simplified continuous location after clustering and the actual location of the user, resulting in higher authenticity of the data. The clustering density ρ represents the number of locations within the same cluster. To some extent, it can replace the distance between locations in clustering. ρ is proportional to *U*. As ρ increases, more actual user locations can be replaced by the clustering centroid, resulting in a larger difference between the simplified distance-based results and the real data.

As the locations with lower access frequencies are reduced before location clustering, the clustering density is minimized. Therefore, the DPLPA can reduce information loss and improve data utility.

## 5. Experimental Results Analysis

### 5.1. Experimental Setting

Our experiments were implemented in Python 3.7 and run on Windows 10 OS, with an Intel Core i7, 3.6 GHz CPU, and 16 GB RAM. The real datasets Geolife [28] and Gowalla [29] were used in our experiments. The Geolife dataset contains 17,621 GPS trajectories of 182 users over three years. Each sample point contains information such as the latitude, longitude, altitude, and time. The dataset contains the user trajectories of a wide range of activities, including traveling home, as well as some recreational and sports activities. The Gowalla dataset is a location-based social network database consisting of 196,591 users and includes 6,442,890 records of users’ behavioral information, including user id, check-in time, latitude, longitude, and location id. Here, only the user id and location id are used.

We compared the DPLPA with the LPPA-PSRDU [22], P-STM [23], LPPM [30], and TLDP [31]. The performance of the proposed algorithm was measured in terms of clustering accuracy, level of privacy protection, data utility, and running time.

### 5.2. Clustering Accuracy

**Figure 6:**
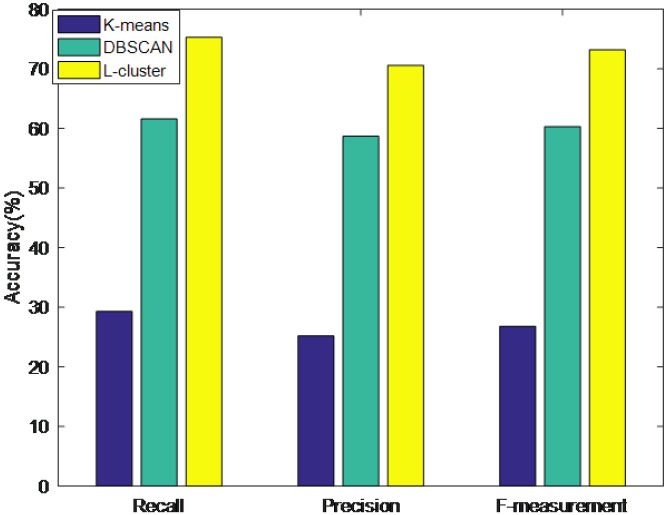
Clustering accuracy.

The clustering accuracy of the L-clustering algorithm was evaluated by comparing the recall, precision, and F-measure of the K-means [32] algorithm and DBSCAN algorithm with those of the L-clustering algorithm, as shown in Figure 6. The precision (*P*), recall (*R*), and F-measure (*F*) were calculated using the following formulas:

(9) $$P=\frac{TP}{TP+FP}$$

(10) $$R=\frac{TP}{TP+FN}$$

(11) $$F=\frac{\left(\alpha^{2}+1\right)P\times R}{\alpha^{2}\left(P+R\right)}$$

where *TP* represents true positives, *FP* represents false positives, and *FN* represents false negatives. The F-measure jointly considers recall and precision, where α is a weight value that adjusts the weight between *P* and *R*.

As shown in the above figure, the L-clustering algorithm exhibited superior performance compared to the K-means and DBSCAN algorithms. The reason for this is that K-means divides the data into k clusters to minimize the sum of the squares of the distance between the data points and their respective clustering centers. However, the algorithm may not perform well for clusters with an arbitrary shape or size. DBSCAN, which groups dense data points and identifies outliers, is able to find clusters with an arbitrary shape and size compared to K-means and is less sensitive to the initial parameter values. However, DBSCAN may not work well with datasets that have varying densities, and it may produce sub-optimal clusters when the data have widely varying densities. The L-clustering algorithm is a density-based clustering algorithm, which identifies high-density core data points and then merges smaller adjacent data points into a larger cluster. L-clustering can process datasets with varying clustering densities and can detect clusters with different shapes and sizes, making it more suitable for the application scenario described in this paper.

### 5.3. Privacy Protection Degree

**Figure 7:**
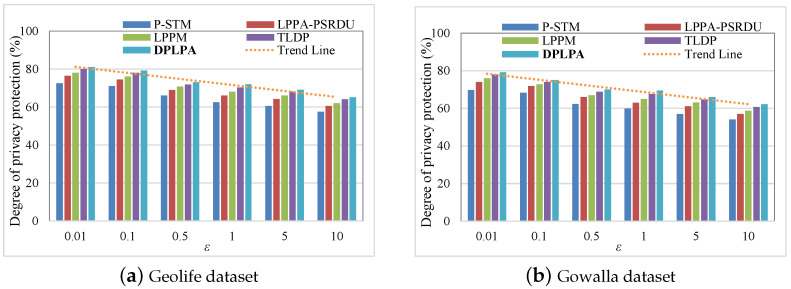
Effect of privacy budget ε on level of privacy protection (ρ = 2, *N* = 200).

**Figure 8:**
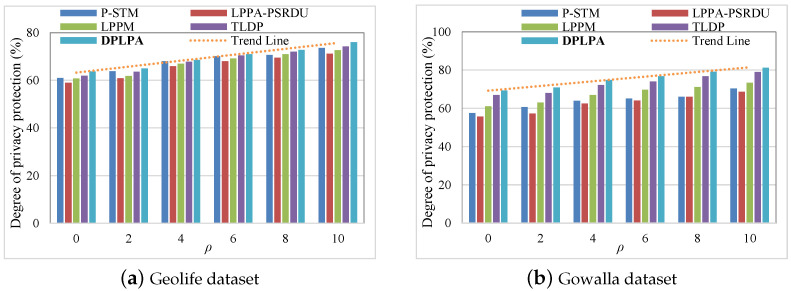
Effect of clustering density ρ on level of privacy protection (ϵ = 2, *N* = 200).

We analyzed the effect of the privacy budget ε, cluster density ρ, and number of locations *N* on the level of privacy protection. The effect of ε on the level of privacy protection was analyzed, and the results are illustrated in a bar chart, as shown in Figure 7. The effect of the clustering density ρ (number of locations per square meter, *N*/m${}^{2}$) on the level of privacy protection was analyzed, as shown in Figure 8.

As seen in Figure 7, the X-axis represents the ε and the Y-axis represents the value of the corresponding level of privacy protection. The dotted yellow line indicates that the level of privacy protection decreased with the increase in the $\varepsilon \in \left\{0.01,0.1,0.5,1,5,10\right\}$, which is inferred from the Laplace probability density function. When the value of ε was the same, the DPLPA obtained the highest value for the level of privacy protection, followed by the TLDP and the P-STM with the lowest. Therefore the DPLPA achieved the highest level of privacy protection, followed by the TLDP and P-STM.

It can be seen in Figure 8 that the level of privacy protection increased with the increase in the ρ (changed from 0 to 10). There was one centroid generated and all the locations were replaced with a unique centroid, enhancing the level of privacy protection. The DPLPA achieved a higher level of privacy protection than the baselines.

**Figure 9:**
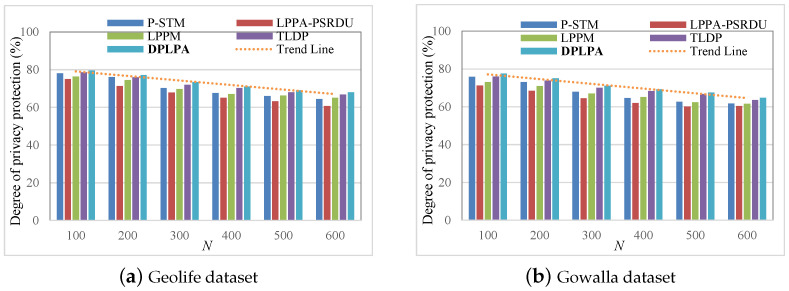
Effect of the number of locations *N* on the level of privacy protection (ϵ = 2, ρ = 2).

Figure 9 shows the levels of privacy protection corresponding to the different numbers of locations *N*. The values of *N* used in the experiments were 100, 200, 300, 400, 500, and 600, respectively. As expected, the level of privacy protection increased with the decrease in *N*, that is, the higher the value of *N*, the lower the level of privacy protection. Because of the higher number of locations, a higher privacy budget was required so more noise was added, thereby reducing the level of privacy protection. Similarly, when the value of *N* was the same, the DPLPA demonstrated the highest level of privacy protection.

### 5.4. Data Utility

**Figure 10:**
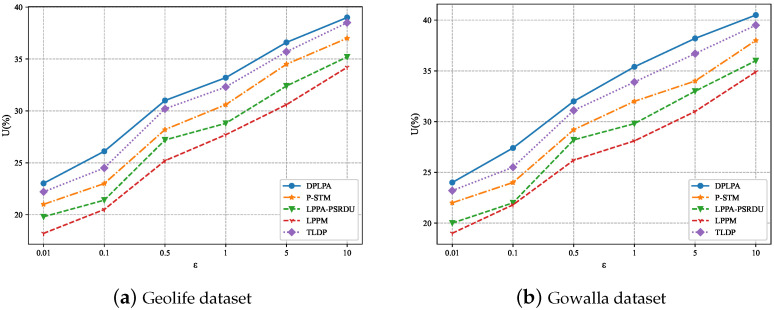
Effect of the privacy budget ε on *U* (ρ = 2, *N* = 200).

By evaluating both data utility and privacy, we can assess how the different methods handled the trade-off between these two aspects. By comparing the DPLPA with the baselines, the advantages of the DPLPA in terms of data utility *U* were evident. The effect of the ε on *U* was analyzed, as shown in Figure 10. *U* increased with the decrease in the ε because with the increase in S, the level of privacy protection decreased and less noise needed to be added, resulting in higher data utility. The data utility of the LPPM was the worst because it considered many factors that affected the location information, resulting in a loss of data integrity. The data utility of the DPLPA was superior compared to the baselines, with minimal error in the distributed position.

**Figure 11:**
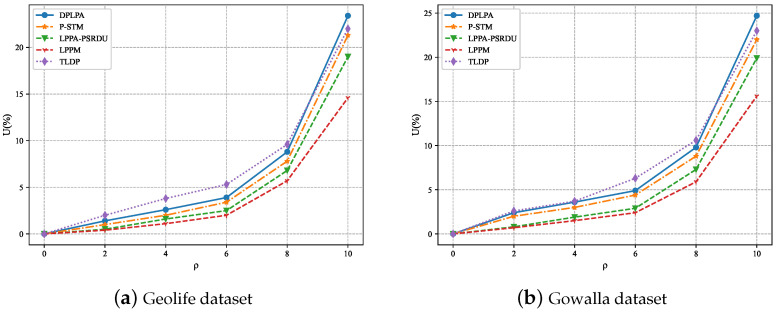
Effect of the privacy budget ρ on *U* (ϵ = 2, *N* = 200).

**Figure 12:**
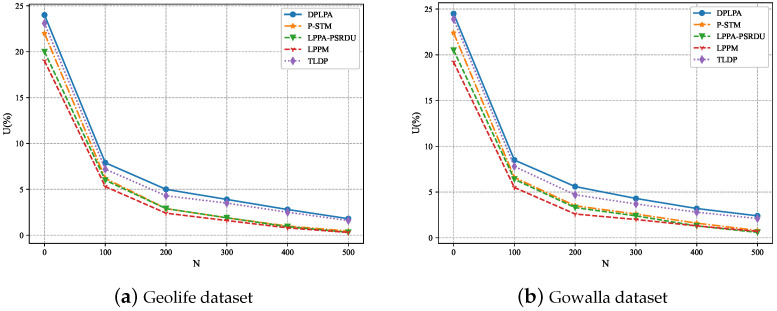
Effect of ρ on *U* (ϵ = 2, ρ = 2).

The effect of ρ on *U* was analyzed, as shown in Figure 11, and the effect of *N* on *U* was also analyzed, as shown in Figure 12. As can be seen, for both datasets, *U* increased with the increase in the ρ and with the decrease in *N*, although the growth rate gradually slowed. The data utility of our method, the DPLPA, was superior to that of the four baselines, regardless of the ρ or *N* values. This is because the DPLPA first eliminated positions with low access frequencies before location clustering, reducing the interference of invalid location information and improving the data utilization rate.

Generally speaking, the proposed method can ensure high data utility while maintaining a high level of privacy. Since data utility can also reflect service quality to some extent, by considering the experimental results in Section 5.3, it can be said that our DPLPA method also had good service performance. Furthermore, we can conclude that the DPLPA provides a favorable trade-off between privacy and data utility for location-based services.

### 5.5. Time Complexity Analysis

**Figure 13:**
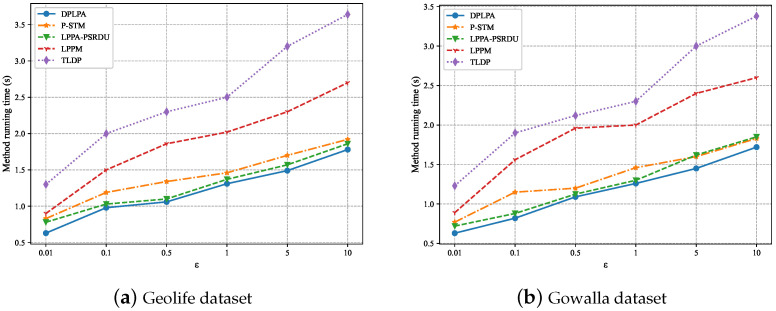
Effect of the privacy budget ε on running time (ρ = 2, *N* = 200).

In this group of experiments, each experiment was executed five times, and the average value was used as the final value. The effect of the privacy budget ε on the running time was analyzed, as shown in Figure 13. The running time of the algorithm increased with the increase in the ε. The larger the ε, the longer it took to allocate the privacy budget and the longer the algorithm’s running time. At the same time, because the DPLPA algorithm only extracted the resident points and added noise, the running time of the DPLPA was the shortest and that of the LPPA-PSRDU algorithm was the longest.

**Figure 14:**
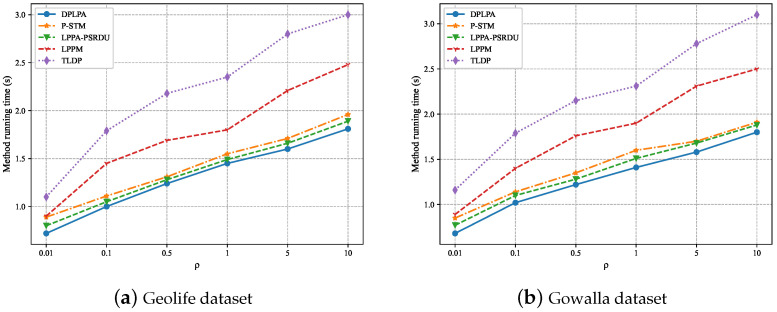
Effect of the clustering density ρ on running time (ϵ = 2, *N* = 200).

The effect of the clustering density ρ on the running time was analyzed, as shown in Figure 14. The experiments showed that for both datasets, the running time increased with the increase in the ρ. The higher the ρ, the longer the running time. The running time of the DPLPA was the shortest and that of the TLDP was the longest, although the TLDP achieved similar performance to the DPLPA in terms of data utility and privacy protection.

**Figure 15:**
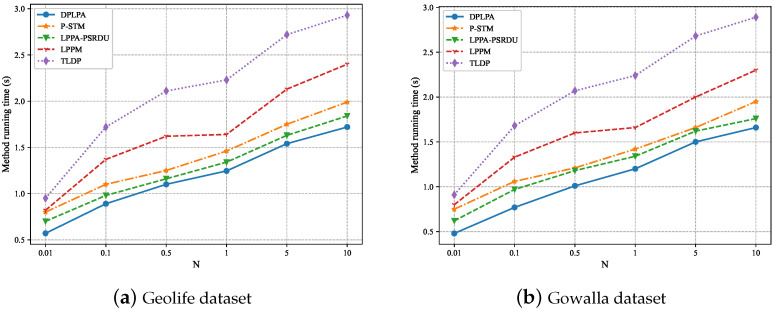
Effect of the number of locations *N* on running time (ϵ = 2, ρ = 2).

Similarly, the effect of the number of locations *N* on the running time was analyzed, as shown in Figure 15. The experiments showed that for both datasets, the running times of the five methods increased with the increase in *N* while remaining within seconds. In this situation, the DPLPA method still had the shortest running time. Despite the fact that the running time of our method showed a gradual growth trend, the trend was relatively gradual. This indicates that the proposed method still has obvious advantages in limited simulation settings.

### 5.6. Location Privacy Protection in Practical Scenarios Based on DPLPA Methods

**Figure 16:**
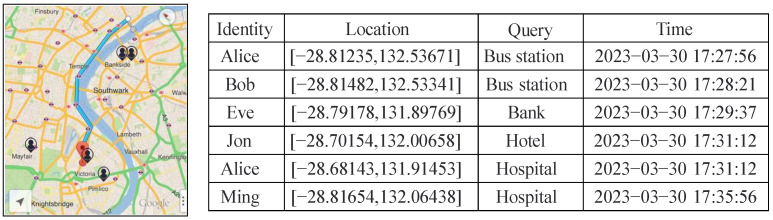
User query information shared with Google Maps based on LBS.

Taking Google Maps as an example, the blue line in the figure below represents the moving trajectory of user Ming. User Ming is represented by a red dot and the other users are represented by black dots. Assuming that there are six users using LBS to query nearby bus stations, banks, hospitals, etc., their shared information, including their current location coordinates (longitude, latitude), query locations, and query times, is shown in Figure 16. It is known that for the first five users, the number of query results for hospital is 1. When the sixth user Ming is added, the number of query results for hospital becomes 2. Therefore, an attacker can infer that the query location for Ming is also hospital.

The DPLPA method proposed in this paper processes sensitive information based on a differential privacy mechanism so that when a user shares their location information, an attacker cannot infer their exact location. Specifically, when a user shares their location information to access certain services, the first step is to extract the user’s resident points, including their long-duration resident points, highly frequented access points, and location points containing sensitive information. Next, multiple groups of continuous locations of the user are simplified and clustered to obtain ROIs. Then, the ROIs are replaced with centroids, and Laplace noise that is suitable for differential privacy is added. As a result, the probability of obtaining specific results through multiple queries is consistent, and the knowledge of an attacker does not change due to the appearance of Ming.

It can be concluded from the above real application scenario that the differential privacy mechanism reduces the risk of an attacker obtaining sensitive information and breaks the connection between identity and location, effectively protecting the privacy of users.

### 5.7. Comprehensive Analysis

Here, we compare the existing works with the proposed DPLPA in terms of privacy protection, data utility, computational overhead, location continuity, and real application scenarios. The results of the comparison are shown in Table 4. From the results, it can be seen that aside from our method, none of these works focused on location continuity. In addition, the proposed DPLPA exhibited good performance. Of course, our method is not perfect and has some limitations that need to be addressed. For example, compared with existing state-of-the-art deep learning methods, the query accuracy of the method proposed in this paper is slightly lower.

## 6. Conclusions

In this paper, we study the privacy protection of continuous location data based on differential privacy and realize differential privacy location protection by constructing ROIs. An L-clustering algorithm is proposed for clustering, which divides the continuous locations into different clusters according to the distance and density, and a cluster centroid is calculated. Then, a location data privacy protection algorithm (DPLPA) is proposed, which allocates a privacy budget to different resident points and centroids and adds Laplace noise to achieve location privacy protection. The experimental results show that the DPLPA can achieve competitive performance in terms of the level of privacy protection, data utilization, and time consumption.

The main contribution of this study is the proposal of an effective method for protecting users’ location privacy for LBS. Compared with other works, the proposed method can effectively ensure the location privacy of users without affecting the efficiency, accuracy, and availability of each LBS query. Therefore, our method is valuable for the protection of user privacy in LBS and can be easily integrated into existing LBS applications, indicating that it can potentially have a positive impact on building privacy-protected LBS applications. However, our work still needs some improvement. For example, due to the diversity of LBS applications, we need to further study how to achieve a connection between our method and each application interface. Furthermore, our approach only considers the privacy protection of users’ continuous historical locations but not their real-time locations. In future work, we will carry out further research on the above problems. 

## Figures and Tables

**Table 1:** Summary of related works.

Reference	Privacy Protection Method	Ideology
[10]	k-anonymity	multi-center clustering; based on geographic semantics
[11]	double k-anonymity	hides location and request information of users
[12]	STA-LPPM	multi-objective particle swarm optimization
[13]	blockchain	anonymous proof for P2P energy-trading location
[14]	DAK k-anonymity	dynamically adjustable by adding and deleting points
[16]	tree structure	attribute-aware privacy-preserving scheme
[17]	Markov model	expands the size of the area
[18]	Markov decision	achieved over an infinite time range
[19]	cluster, k-anonymity	groups users by learning their trajectory data
[20]	graph trajectory data	measurement method based on edges and nodes
[21]	differential privacy	adds random noise and gathers the centroids of clusters
[22]	personalized security	considers users’ historical GPS trajectory and attributes
[23]	P-STM	mines social connections of users’ trajectories
[24]	DP, cluster	constructs continuous time regions
[25]	PrivSem	combines k-anonymity, l-diversity, and DP

**Table 2:** Original continuous location data.

ID	Continuous Position
1	a→b→d→f→h→j→l
2	a→f→i→j→r→s→w
3	a→i→j→r→s→w
4	a→i→o→v→w
5	a→g→o→r→w
6	a→g→i→o→v→r→w

**Table 3:** Original continuous location data.

ID	Location	Frequency	ID	Location	Frequency
1	a	6	2	f	6
3	g	2	4	h	l
5	i	4	6	j	3
7	o	3	8	q	l
9	r	4	10	s	2
11	v	2	12	w	6
13	s	l			

**Table 4:** Comparative Summary.

Function/Reference	[11]	[12]	[14]	[15]	[16]	[23]	[24]	[25]	DPLPA
Privacy protection	✓	✓	✓	✓	✓	✓	✓	✓	✓
Data utility	✓	✓	✕	✓	✕	✓	✓	✕	✓
Computing overhead	✕	✕	✓	✕	✓	✕	✓	✓	✓
Location continuity	✕	✕	✕	✕	✕	✕	✕	✕	✓
Real scenario	✕	✓	✓	✕	✕	✕	✕	✕	✓

## Data Availability

The data are contained within the article.
